# The lateral patellar retinaculum is thicker in paediatric and adolescent patients with patellofemoral instability: An MRI analysis

**DOI:** 10.1002/jeo2.70202

**Published:** 2025-04-01

**Authors:** Danielle E. Chipman, Emilie Lijesen, Peter M. Cirrincione, Danielle S. Gorelick, Shae K. Simpson, Douglas N. Mintz, Daniel W. Green

**Affiliations:** ^1^ Department of Paediatric Orthopedics Hospital for Special Surgery New York New York USA; ^2^ Department of Radiology Hospital for Special Surgery New York New York USA

**Keywords:** lateral patellar retinaculum, medial patellofemoral ligament reconstruction, patellar instability, patellar tilt, paediatrics

## Abstract

**Purpose:**

The purpose of this study was to examine the thickness of the lateral patellar retinaculum (LPR) and patellar tilt in paediatric and adolescent patients who undergo a medial patellofemoral ligament (MPFL) reconstruction. The authors hypothesise that patients undergoing MPFL reconstruction will have a thicker LPR and increased patellar tilt when compared to a comparison cohort.

**Methods:**

Preoperative magnetic resonance imaging (MRI) of patients ≤ 18 years old who underwent an MPFL reconstruction was retrospectively reviewed. Patients were included if they had a proton density preoperative axial MRI performed internally at our institution. Included patients were matched to a comparison cohort. LPR thickness and patellar tilt were measured on MRI. LPR thickness and patellar tilt were compared between the MPFL cohort and the comparison cohort.

**Results:**

A total of 363 patients were identified. 145 participants were successfully matched to the comparison cohort. The mean age in the MPFL cohort was 14.4 ± 2.0 years and 68% were female. The LPR thickness in the MPFL cohort was significantly greater than the LPR thickness in the comparison cohort (*p* < 0.001). The patellar tilt was significantly greater in the MPFL cohort compared to the control cohort (*p* < 0.001). There was no statistical difference in patients undergoing MPFL reconstruction and the occurrence of a lateral release/lengthening procedure.

**Conclusion:**

The LPR was significantly thicker on preoperative MRI in patients undergoing MPFL reconstruction compared to a comparison cohort, indicating that increased LPR thickness is a potential marker of patellofemoral instability on imaging.

**Level of Evidence:**

Level III.

**Abbreviations**
LPR: lateral patellar retinaculum
MPFL: medial patellofemoral ligament
MRI: magnetic resonance imaging
TTO: tibial tubercle osteotomy

## INTRODUCTION

Patellar instability and dislocation is a traumatic injury that often requires surgery and can have detrimental impacts on patient quality of life [1, 7]. Paediatric and adolescent patients who undergo a medial patellofemoral ligament (MPFL) reconstruction for surgical treatment of patellar instability occasionally undergo a concomitant release of the lateral patellar retinaculum (LPR). As the LPR is crucial for patellar stability, releasing the LPR when too tight improves the patella's alignment and positioning in the trochlear groove [15]. The LPR also has more nerve fibre content and a higher number of elastic fibres compared to knee ligaments such as the MPFL [2].

Limited literature has described characteristics of the LPR in conjunction with undergoing an MPFL reconstruction. The LPR has been described as having more loose connective fibres than the medial patellar retinaculum, thought to aid in patellar stability by reducing the tension between the patella and lateral compartment of the knee [8]. Cadaver studies have shown the LPR to be a stabilising knee structure but lack specific information regarding LPR characteristics and its relationship to patellar instability [8, 13]. The authors are unaware of any studies assessing LPR thickness in patients undergoing an MPFL reconstruction. Patellar tilt is also a commonly used radiographic measure for assessing patellar instability, and thus, we were interested in assessing the relationship between patellar tilt and LPR thickness [14].

The purpose of this study was to examine the thickness of the LPR and patellar tilt in paediatric and adolescent patients who undergo an MPFL reconstruction. The authors hypothesise that patients undergoing an MPFL reconstruction will have a thicker LPR and increased patellar tilt when compared to controls.

## METHODS

**Figure 1:**
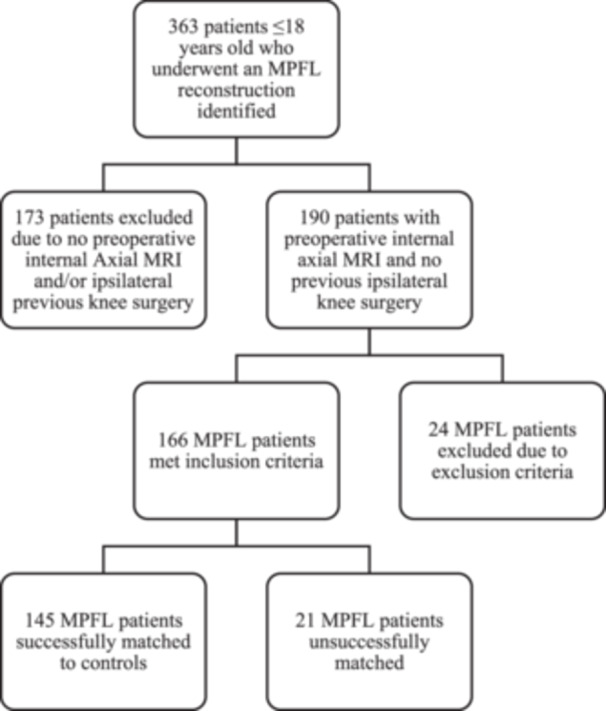
Diagram demonstrating inclusion and exclusion criteria for final cohort. MPFL, medial patellofemoral ligament; MRI, magnetic resonance imaging.

After Institutional Review Board approval, a review of preoperative magnetic resonance imaging (MRI) of patients ≤ 18 years old who underwent an MPFL reconstruction by a single surgeon at a single institution between 2016 and 2022 was performed. Patients were excluded if they had a history of previous ipsilateral knee surgery, were syndromic, and/or had obligatory patellar dislocation in flexion [12]. Patients were included if they had a proton density preoperative axial MRI performed internally at our institution. Included patients were matched to a comparison (Control) cohort based on age within 1.5 years at time of imaging, sex, and laterality (Figure 1).

### MRI measures

**Figure 2:**
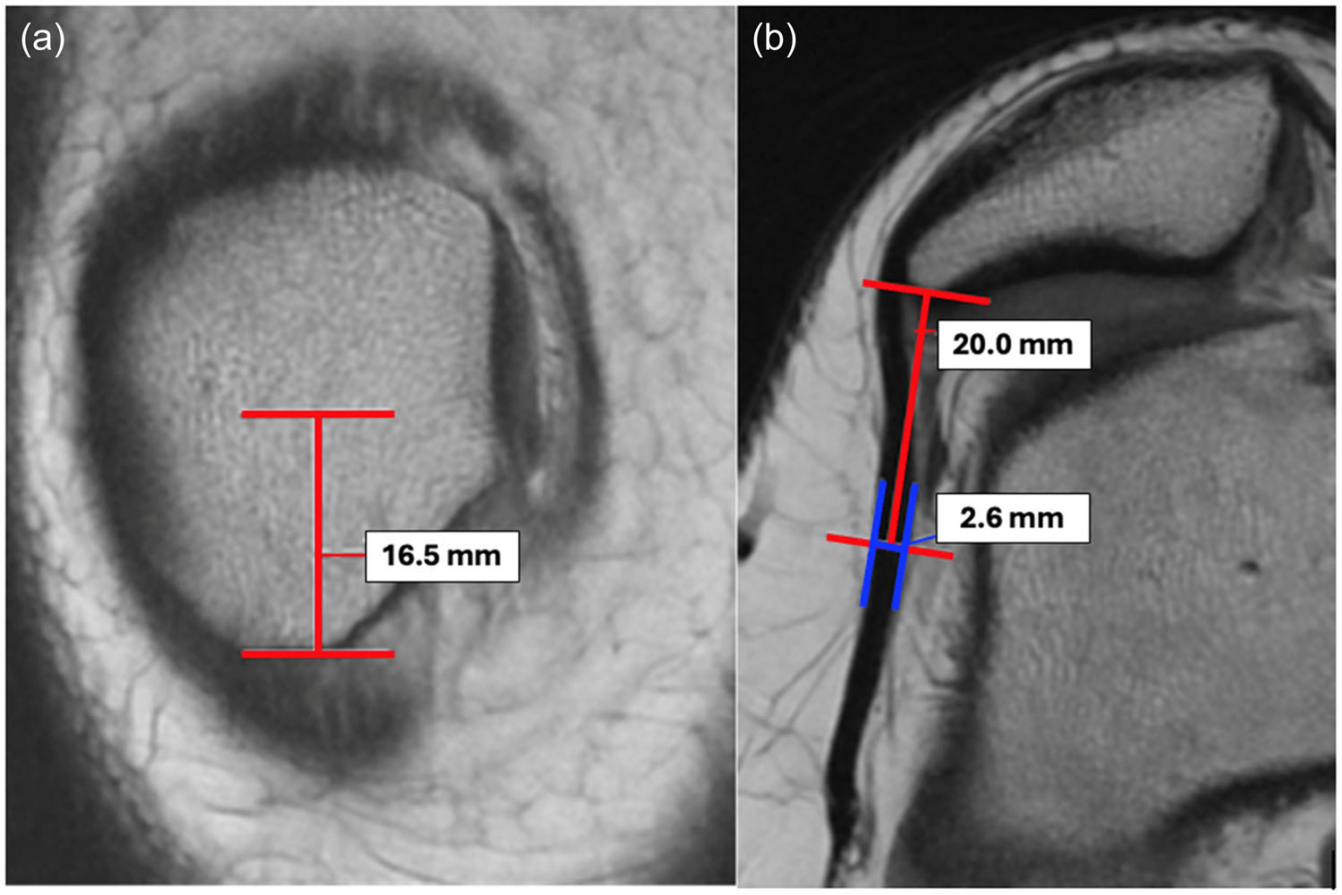
LPR Measurement on MRI. (a) Coronal MRI slice with longest height of patella identified, distance measured and divided by half to identify the midpoint (ex: 16.5 mm). (b) Midpoint from coronal view used to identify slice on axial view, 20 mm measured from lateral edge of patella to LPR, at this point LPR thickness is measured (ex: 2.6 mm). LPR, lateral patellar retinaculum; MRI, magnetic resonance imaging.

LPR thickness was measured on an axial proton density preoperative MRI. First, the longest height of the patella was found on the coronal MRI. The height was measured and divided in half on the coronal MRI to identify the midpoint. Next, using the localiser, the midpoint of the patellar height was identified on the axial MRI. Twenty millimetres (mm) was measured from the lateral edge of the patella to the LPR. The thickness of the LPR was measured at this point (Figure 2). The LPR thickness measurements were performed by two blinded raters. Two‐way mixed effects intraclass correlation coefficients (ICC) were calculated for interrater reliability of the LPR thickness measurement. Additionally, patellar tilt was measured on preoperative MRIs. Demographic and surgical data was collected for all included patients undergoing MPFL reconstruction.

### Statistical analysis

An independent samples Mann–Whitney *U* test was performed to compare LPR thickness between the MPFL cohort and the comparison cohort. Statistical significance was set to *p* ≤ 0.05. IBM SPSS Statistics version 22 for Windows was used for all statistical analyses.

## RESULTS

A total of 363 patients were identified. A total of 145 patients were successfully matched to the comparison cohort. The mean age of the 145 patients in the MPFL cohort was 14.4 ± 2.0 years (range: 8.5–18.9 years) and 68% were female (Table 1). Height and weight at time of imaging were also compared between cohorts (height: MPFL 165.5 ± 10.6 vs. control 161.6 ± 12.0, *p* = 0.004; weight: MPFL 60.6 ± 17.0 vs. control 54.7 ± 13.8, *p* = 0.001). In the MPFL cohort, 85% received an autograft and 99% were reconstructed with a hamstring graft. 15% of patients undergoing MPFL reconstruction underwent a concomitant tibial tubercle osteotomy (TTO) (Table 2).

**Table 1:** Demographics of patients in MPFL and control cohort.

	All patients (*N* = 290)	MPFL cohort (*N* = 145)	Control cohort (*N* = 145)	*p* value
	Mean ± SD			
Age at imaging (years)	14.2 ± 2.1	14.4 ± 2.0	14.1 ± 2.1	0.059
	*N* (%)			
Sex				
Female	196 (68)	98 (68)	98 (68)	
Male	94 (32)	47 (32)	47 (32)	

Abbreviations: MPFL, medial patellofemoral ligament; SD, standard deviation.

**Table 2:** Surgical details of patients in MPFL cohort.

	*N* (%)
Autograft/allograft	
Autograft	123 (85)
Allograft	22 (15)
Graft type	
Hamstring	143 (99)
Quad	2 (1)
TTO	22 (15)
Lateral release/lengthening	34 (23)

Abbreviations: MPFL, medial patellofemoral ligament; TTO, tibial tubercle osteotomy.

**Figure 3:**
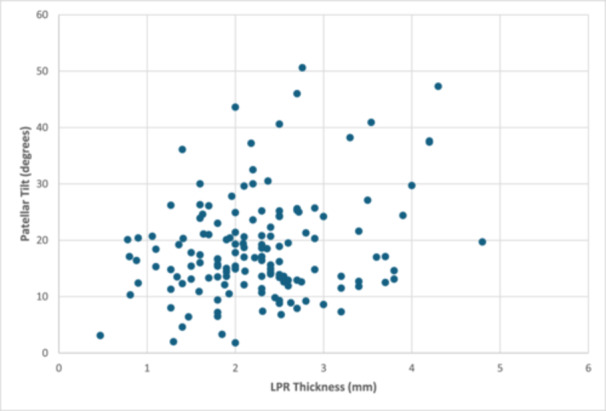
Correlation between patellar tilt and LPR thickness for patients in MPFL cohort. LPR, lateral patellar retinaculum; MPFL, medial patellofemoral ligament.

The LPR thickness in the MPFL cohort was significantly greater than the LPR thickness in the comparison cohort (2.3 ± 0.8 mm vs. 1.9 ± 0.6 mm, *p* < 0.001). The patellar tilt was significantly greater in the MPFL cohort compared to the control cohort (18.5 ± 9.2 degrees vs. 6.9 ± 4.7 degrees, *p* < 0.001) (Table 3). There was no statistical difference in patients undergoing MPFL reconstruction and the occurrence of a lateral release/lengthening procedure (Table 4). There was a significant (*p* < 0.001) weak positive correlation (0.270) between LPR thickness and patellar tilt in the MPFL cohort (Figure 3). The LPR thickness ICC was 0.990.

**Table 3:** LPR thickness and patellar tilt between MPFL and control cohort.

	MPFL cohort	Control cohort	*p* value
	Mean ± SD		
LPR thickness (mm)	2.3 ± 0.8 (0.5–4.8)	1.9 ± 0.6 (0.8–3.9)	<0.001*
Patellar tilt (degrees)	18.5 ± 9.2 (1.8–50.6)	6.9 ± 4.7 (−6.7 to 19.7)	<0.001*

Abbreviations: LPR, lateral patellar retinaculum; MPFL, medial patellofemoral ligament; SD, standard deviation.

**Table 4:** Lateral release/lengthening rate for patients in MPFL cohort.

	LPR thickness	*p* value
	Median (IQR)	
Lateral release/lengthening (*n* = 34)	2.2 (1.0)	0.998
No lateral release/lengthening (*n* = 116)	2.3 (0.9)	

Abbreviations: IQR, interquartile range; LPR, lateral patellar retinaculum; MPFL, medial patellofemoral ligament.

## DISCUSSION

The most important finding of this study was that patients undergoing an MPFL reconstruction had a significantly thicker LPR when compared to a control cohort. There was a weak positive correlation between patellar tilt and LPR thickness in patients undergoing MPFL reconstruction. There was no statistical difference in LPR thickness for the MPFL cohort based on whether a lateral release/lengthening was performed. Overall, increased patellar tilt and LPR thickness were significantly higher in the MPFL cohort, suggesting both to be imaging parameters associated with MPFL reconstruction in patients with patellofemoral instability.

The LPR is located on the anterolateral aspect of the patellofemoral joint and extends from the lateral side of the patella [15]. Providing stability and support for the patella, lateral patellar instability is often clinically thought to be linked to a tight LPR, whereby a lateral release/lengthening may be indicated to improve the clinical success of MPFL and patellar stabilisation surgery. Controversy remains regarding the ease of visualising the LPR on MRI, however, its structure has been informative of lateral release/lengthening to improve patellar instability [6].

As this study demonstrated that the LPR was significantly thicker in patients undergoing an MPFL reconstruction when compared to controls, we speculate LPR thickness may be an indicator of patellofemoral instability. Lateral release/lengthening has commonly been used at our institution to address patellar instability in conjunction with an MPFL reconstruction, however, there was no significant difference in performing a lateral release based on LPR thickness. We speculate thicker LPRs may have a greater influence on patellar instability by creating a stronger pull of the patella to the lateral side of the knee due to its increased thickness, as a tight lateral retinaculum has previously been associated with patellar instability [6]. This further supports our statistical findings of increased LPR thickness, for which we speculate LPR thickness to be linked to operative treatment of patellofemoral instability.

This study also found that patellar tilt was significantly greater in patients undergoing MPFL reconstruction compared to the Control cohort, consistent with prior literature and indications for an MPFL reconstruction. Patellar tilt in the MPFL reconstruction group was also beyond the normal value of 15 degrees [9]. Increased patellar tilt is a highly specific and reliable radiographic indicator for patellar instability [3, 4, 5, 10]. A study by Pascual‐Leone et al. [11] demonstrated that MPFL reconstruction has been shown to improve patellar tilt. Further, our work observed a significant (weak positive) correlation between patellar tilt and LPR thickness. We are unaware of any studies theorising this association; however, we speculate that increased LPR thickness may increase patellar tilt by increasing tension between the lateral side of the patella and the lateral knee compartment. As the LPR has been shown to reduce tension load on the patella, increased thickness may also produce a stronger force on the lateral aspect of the patella, causing a greater tilt [8]. Future studies assessing the composition of the LPR may be warranted to assess how thickness directly relates to patellar tilt.

This study is not without limitations. First, patients with single, recurrent, and fixed dislocations were included, and therefore the number of dislocations may have impacted whether a patient was recommended for an MPFL reconstruction. This does not account for the relationship between LPR thickness and the number of dislocations which may have been a confounding variable. Second, this study included patients with and without isolated MPFL reconstructions, whereby abnormal patellofemoral morphology may have influenced any additional bony procedures impacting LPR thickness and/or patellar tilt. Further, patients were excluded if they experienced any trauma causing patellar instability‐imposed damage seen on MRI, as this rendered the MRI unreadable and would affect measurement accuracy. Third, as this study was performed using patients treated by one orthopaedic surgeon, there may be inescapable bias in the decision to perform surgery. Fourth, we were unable to control the differences in height and weight between our cohorts. Patients with higher body‐mass index (BMI) are at an established higher risk of MPFL tear, naturally increasing the overall height and weight of our MPFL cohort. Although these values exhibit statistically significant differences, the absolute difference is quite small. However, the authors believe these limitations do not negate the findings of this study as it involved a large cohort of patients and MPFL reconstruction is a widely used treatment option for patients with patellar instability. Finally, this is the first study to define LPR thickness as a marker of patellar instability. While we believe these findings are important, their novelty makes future studies necessary to truly define a meaningful cutoff of LPR thickness indicative of patellar instability.

## CONCLUSION

This study demonstrated that the LPR was significantly thicker on preoperative MRI in patients undergoing MPFL reconstruction compared to a comparison cohort. This is one of the first studies to demonstrate that LPR thickness is associated with pathologic patellofemoral instability on imaging.

## AUTHOR CONTRIBUTIONS

Danielle E. Chipman, Peter M. Cirrincione, Danielle S. Gorelick, Douglas N. Mintz and Daniel W. Green developed the methodology of this study. Danielle E. Chipman, Emilie Lijesen and Daniel W. Green drafted the manuscript. Danielle E. Chipman, Emilie Lijesen, Peter M. Cirrincione, Danielle S. Gorelick, Douglas N. Mintz and Daniel W. Green edited and approved the final draft of the manuscript. Shae K. Simpson collected data and edited the manuscript to help with revisions.

## CONFLICT OF INTEREST STATEMENT

Danielle E. Chipman, Emilie Lijesen, Peter M. Cirrincione, Danielle S. Gorelick and Shae K. Simpson declare no conflict of interest. Douglas N. Mintz is a board or committee member for American College of Radiology, New York State Radiological Society, and Society of Skeletal Radiology. Daniel W. Green is a consultant for Arthrex Inc. and receives royalties for Arthrex Inc. and Orthopediatrics. Daniel W. Green receives hospitality payments from Orthopediatrics and receives faculty/speaker fees from Synthes.

## ETHICS STATEMENT

This study was reviewed and approved by the Hospital for Special Surgery Institutional Review Board (IRB #2022‐1725). Not required for this study, as it was retrospective.

## Data Availability

The data for this study is not publicly available, however, requests can made to view data if reasonable request.
